# The impact of analytical cognitive style on business model innovation in new ventures: The moderating role of self-efficacy and environmental uncertainty

**DOI:** 10.1371/journal.pone.0335256

**Published:** 2025-10-24

**Authors:** Li Hui, Zhang Xuebing

**Affiliations:** Business School, Shandong Xiehe University, Jinan, China; Universiti Pertahanan Nasional Malaysia, MALAYSIA

## Abstract

In the context of digital-intelligent transformation, the deep integration of data elements has reshaped the cognitive boundaries of entrepreneurial decision-making. New ventures that leverage rational, data-driven analysis to guide strategic choices can transcend the bounded rationality of traditional experiential decision-making, thereby enhancing operational efficiency, market competitiveness, and long-term sustainability. Drawing on a social cognitive perspective, this study empirically examines survey data from 138 start-up firms to investigate the impact of analytical cognitive style on business model innovation in new ventures. Results indicate that analytical cognitive style is positively associated with both efficiency-oriented and novelty-oriented types of BMI. Moreover, entrepreneurial self-efficacy positively moderates the relationship between analytical cognitive style and efficiency-oriented BMI, while negatively moderating the relationship between analytical cognitive style and novelty-oriented BMI. Additionally, environmental uncertainty negatively moderates the link between analytical cognitive style and novelty-oriented BMI. These findings provide meaningful theoretical insights into the cognitive foundations of BMI and offer practical guidance for entrepreneurs seeking to innovate under conditions of uncertainty.

## 1. Introduction

“How new ventures overcome liabilities of newness” has long been a central concern in entrepreneurship research. Studies show that new ventures can use business model innovation to recombine resources efficiently, reduce operational costs, and improve profitability [1].

Since the late 20th century, research has shifted from behavioral rationality to cognitive rationality [2]. Scholars have focused on entrepreneurs’ heuristic cognitive shortcuts [3], self-serving biases in attribution [4], and tendencies toward over-optimism and overconfidence[5,6]. Consequently, the cognitive perspective has become mainstream in business model research. Studies show that entrepreneurs with intuitive and heuristic cognitive styles are more likely to design highly innovative business models [7]. However, next-generation digital technologies such as big data, AI, blockchain, and cloud computing have become critical production factors in the digital economy. Data-driven business model innovation has thus gained practical attention. This trend calls for theoretical research to expand the traditional cognitive perspective and to examine the role of analytical cognitive styles, which contrast with intuitive and heuristic approaches.

Analytical cognitive style emphasizes rational analysis and systematic decision-making. While a few scholars have considered its influence on BMI, the construct is often treated as a secondary variable. Martins et al. [8] explored how categorical reasoning and conceptual combination enable entrepreneurs to generate innovative business models. Wang et al. [9] analyzed the role of rational cognitive style in facilitating BMI via entrepreneurial resilience. Scholars in the big data domain argue that reliance on intuition under conditions of limited information often leads to sub-optimal decisions, whereas data-driven strategies provide more accurate and comprehensive support for identifying BMI opportunities [10,11].

Although prior research has examined the influence of analytical cognitive style on business model innovation, it has largely overlooked key questions, such as under what conditions this relationship is stronger and how it varies across different types of business model innovation. Consequently, the mechanisms through which analytical cognitive style affects business model innovation remain underexplored.

To address these gaps, this study integrates insights from social cognitive theory and strategic management theory. Using data from Chinese start-ups, we investigate the differential relationships between analytical cognitive style and various types of business model innovation, and examine the moderating effects of entrepreneurs’ self-efficacy and environmental uncertainty. China is currently one of the most dynamic regions for global innovation and entrepreneurship, making the findings particularly representative. This study contributes to theory by extending the cognitive perspective of business model research to the digital economy context and clarifies the boundary conditions of analytical cognitive style’s impact. Practically, the results provide guidance for new ventures to pursue business model innovations more effectively and mitigate innovation failure risks.

## 2. Theoretical analysis and research hypotheses

### 2.1. Theoretical foundation

#### 2.1.1. Cognitive style.

Cognitive style is a foundational concept in psychology, with its earliest academic exploration tracing back to the 1940s. It refers to individuals’ consistent preferences in gathering, processing, and evaluating information [12]. Contemporary scholars typically distinguish cognitive styles based on the dual-process theory of human cognition—rational versus subjective tendencies. Notable models include Kirton’s [13] Adaption–Innovation theory and Allinson and Hayes’ [12] intuitive–analytical model, which synthesizes insights from 29 existing frameworks. Sadler-Smith et al. [14] argue that these models converge conceptually, as their polar dimensions express equivalent cognitive traits. Among them, the intuitive–analytical model is more widely adopted in entrepreneurship research and is therefore employed in this study.

An intuitive cognitive style emphasizes heuristics and experiential reasoning, while an analytical cognitive style favors systematic, logical, and stepwise information processing [15]. In entrepreneurship contexts, decision-making is often dominated by intuitive reasoning, as entrepreneurs rely on prior knowledge, analogical thinking, and heuristics [16,17]. This is because individuals tend to adopt cognitive styles that match their operating environments [12], and entrepreneurial settings—characterized by ambiguity, dynamism, complexity, and uncertainty—tend to align more closely with intuitive reasoning [18].

However, over-reliance on intuition can lead to cognitive biases and poor decision-making outcomes [19,20]. According to the cognitive continuum theory, analytical and intuitive reasoning lie on opposite ends of a dynamic cognitive spectrum, with individuals oscillating between them based on situational demands [21]. Successful entrepreneurs do not rigidly adhere to one cognitive mode but rather shift their approach in response to changing environmental cues. The entrepreneurial environment is highly VUCA, characterized by volatility, uncertainty, complexity, and ambiguity. Nevertheless, the continuously evolving data factor market provides a foundation for rational analysis, making it both feasible and highly promising to leverage analytical cognitive styles for data-driven innovation and decision-making.

#### 2.1.2. Business model innovation.

A business model is a unique configuration of transactions that generates value for a firm and its stakeholders—not merely through activity choice, but through a logic that links value creation with value capture [22,23]. Business model innovation (BMI) is a relative concept, referring to deviation from a baseline model. It applies both to incumbents reconfiguring their models and startups designing novel configurations ab initio [24]. Amit and Zott [22] identify four archetypes of BMI: efficiency, novelty, lock-in, and complementarity. Of these, Zott and Amit [25] emphasize efficiency-oriented and novelty-oriented BMI as foundational dimensions and develop corresponding measurement instruments. These constructs have since become widely accepted in the literature and guide the empirical framework of this study.

BMI in startups reflects how entrepreneurs cognitively construct innovative business configurations to guide firm operations. A cognitive perspective on BMI posits that it emerges from entrepreneurs’ internal cognitive processes and mental schema formation [26,27]. For instance, Battistella et al. [28] highlight how entrepreneurs construct meaning by interpreting external cues, generating schematic knowledge. Martins et al. [8] elaborate how, in stable environments, entrepreneurs engage in analogical reasoning and conceptual blending to design novel business models. As cognition is shaped by cognitive style, variation in style explains heterogeneity in BMI outcomes [29]. However, the relationship between cognitive style and BMI remains underexplored. Furthermore, entrepreneurial self-efficacy and environmental uncertainty represent core individual and contextual contingencies. Understanding their moderating effects is crucial to revealing how cognitive styles translate into innovative business models.

### 2.2. Research hypotheses

#### 2.2.1. Analytical cognitive style and BMI.

Entrepreneurial cognitive style is a key determinant of strategic decisions [30]. Individuals with an analytical style prefer structured, logic-based decision-making supported by objective data [12], a trait highly relevant to BMI.

In efficiency-oriented BMI, analytical reasoning helps entrepreneurs identify inefficiencies and construct multidimensional data models for predictive insight. For instance, Amazon improved inventory turnover by 40% through customer behavior analytics. Wang et al. [9] found that firms leveraging analytical tools increased market responsiveness by 28.6% on average. In novelty-oriented BMI, analytical styles also enable breakthrough innovation by deconstructing existing logic and reconfiguring value propositions. Uber used data analytics to reconstruct ride-matching mechanisms, and Netflix leveraged viewing data to evolve into a content producer. Even Tesla reimagined energy models in electric vehicles through battery data analysis.

Consistent with cognitive-environment fit theory, data-rich contexts amplify the innovation-enabling effects of analytical styles. As digital transformation accelerates, such cognitive modes are increasingly advantageous. Based on the above analysis, this paper proposes the following hypotheses:

*Hypothesis 1a: Analytical cognitive style is positively associated with efficiency-oriented BMI.*

*Hypothesis 1b: Analytical cognitive style is positively associated with novelty-oriented BMI.*

#### 2.2.2. The moderating role of entrepreneurial self-efficacy.

Entrepreneurial self-efficacy refers to an individual’s belief in their ability to accomplish specific entrepreneurial tasks and achieve goals [31]. Within entrepreneurial contexts, self-efficacy shapes managerial risk preferences, decision-making speed, and approaches to resource orchestration. According to social cognitive theory, high self-efficacy leads entrepreneurs to perceive greater control over their environment. However, the influence of self-efficacy may vary depending on the type of innovation involved [32].

Entrepreneurs with an analytical cognitive style tend to make decisions based on systematic assessments and risk evaluations [12]. While such thoroughness enhances decision quality, it can also delay execution. High self-efficacy can mitigate these delays by increasing confidence in analytical outcomes. Entrepreneurs with high self-efficacy are more likely to act decisively based on their analysis, reducing decision latency and improving resource efficiency [33]. Moreover, their confidence enhances their ability to persuade team members to adopt their proposed solutions, accelerating consensus and implementation [34]. For example, under lean startup models, self-efficacious entrepreneurs drive rapid deployment of standardized processes, lowering trial-and-error costs.

However, in the context of radical innovation, high self-efficacy may have adverse effects. Entrepreneurs who are highly confident may overly rely on prior success, falling into a “capability trap” that prioritizes exploiting existing resources rather than exploring new models [George & Bock, 2012]. Kodak’s delayed digital transformation exemplifies such overconfidence. Additionally, these entrepreneurs may underestimate the uncertainty of innovation environments, discount external feedback, and suppress the search for novel ideas. Jiang et al. [35] find that excessive self-efficacy reduces information scanning and impedes the generation of breakthrough innovations. Aspara et al. [36] also note that overconfidence in resource-based advantages limits experimentation with new transactional models. Based on the above analysis, this paper proposes the following hypotheses:

*Hypothesis 2a: Entrepreneurial self-efficacy positively moderates the relationship between analytical cognitive style and efficiency-oriented BMI.*

*Hypothesis 2b: Entrepreneurial self-efficacy negatively moderates the relationship between analytical cognitive style and novelty-oriented BMI.*

#### 2.2.3. The moderating role of environmental uncertainty.

Environmental uncertainty refers to the degree of unpredictability and turbulence in a firm’s external environment, including policy shifts, demand volatility, technological disruption, and competitive reconfigurations. According to dynamic capabilities theory, firms must adjust decision logics and resource deployment strategies under uncertainty to sustain competitive advantage.

In high-uncertainty contexts, firms face greater resource constraints, market instability, and competitive threats. Efficiency-oriented BMI provides a more viable survival strategy than radical innovation under such conditions. Entrepreneurs with analytical cognitive styles rely on evidence-based decision-making, leveraging market data and process diagnostics to identify performance bottlenecks and improve cost efficiency [37]. Empirical evidence suggests that firms emphasizing analytical efficiency often outperform competitors in cost-sensitive environments. Conversely, stable conditions allow greater resource slack and encourage risk-taking, which aligns with exploration strategies. Christensen’s [38] disruptive innovation theory argues that incumbents in stable environments often deprioritize efficiency to pursue growth through experimentation. Thus, environmental uncertainty strengthens the relationship between analytical cognition and efficiency-oriented BMI.

Novelty-oriented BMI, by contrast, demands the reconfiguration of existing industry logic, transaction structures, and revenue models [22]. Analytical entrepreneurs prefer clearly defined causal pathways and robust datasets, yet in highly uncertain environments, information is ambiguous and fast-changing. Such volatility inhibits structured exploration. From a resource-based perspective, novel business models require distinctive and often irreversible resource commitments, which are riskier under uncertainty and exacerbate decision aversion [Kahneman & Tversky , 1979]. [39 ]found that under turbulent conditions, organizations gravitate toward incremental improvements rather than transformative change. In contrast, stable environments lower feedback ambiguity and experimentation costs, encouraging entrepreneurs to pursue bold innovations. Based on the above analysis, this paper proposes the following hypotheses:

*Hypothesis 3a: Environmental uncertainty positively moderates the relationship between analytical cognitive style and efficiency-oriented BMI.*

*Hypothesis 3b: Environmental uncertainty negatively moderates the relationship between analytical cognitive style and novelty-oriented BMI.*

## 3. Research design

### 3.1. Sample selection

This study selects entrepreneurs of newly founded firms as the research subjects. To maximize the generalizability of the findings, no restrictions were imposed on the industry or size of the sampled firms. Following Zahra et al. [40], newly founded firms are defined as enterprises established within the past six years. Regarding the selection of survey regions, this study combined the “China Innovation and Entrepreneurship Index” with available research resources, and chose Beijing, Tianjin, Hebei Province, and Liaoning Province as the survey areas.

Given the difficulty of obtaining time-series data on entrepreneurs’ analytical cognitive style and self-efficacy, this study adopts cross-sectional observational data for measurement and analysis. The research questionnaire was independently designed by the research team and revised based on more than 20 pilot surveys prior to the formal investigation, in order to ensure the scientific rigor and comprehensibility of the items.

In the formal survey stage, the research team commissioned a qualified professional market research company to distribute and collect the questionnaires. The company selected enterprises that met the sampling criteria from its client database, and trained interviewers conducted one-on-one on-site visits. Prior to each interview, the company obtained consent from the enterprise managers. During the survey, interviewers explained the purpose and content of the study to respondents, and only proceeded after their consent was obtained. The interviews were conducted in a question-and-answer format, with responses recorded item by item. Respondents had the right to withdraw at any stage, and any contact details or business cards provided during the process were used solely for authenticity verification and were not disclosed in the study findings. To ensure data collection quality and reliability, the research team accompanied and monitored approximately 30% of the interviews. All participants provided verbal informed consent, which was recorded and witnessed by the interviewers. This procedure was reviewed and approved by the Academic Ethics Committee of **Shandong Xiehe University**. No minors were involved in this study.

The questionnaire distribution and collection lasted three months, during which 214 questionnaires were distributed, and 145 were initially returned as valid, yielding a response rate of 67.76%. To ensure data quality, the research team conducted strict screening of all returned questionnaires, excluding those with contradictory answers, inconsistencies in cross-check items, extensive missing responses, or patterned answers, and further verified responses through telephone follow-ups. Ultimately, 138 valid questionnaires were confirmed, with a final effective response rate of 64.49%. According to the criteria of Rea and Parker [41], although the sample size is relatively modest, it exceeds five times the number of variables in the model and is greater than 100, thereby meeting the basic requirements for empirical research. Descriptive statistics of the sample characteristics are presented in Table 1.

**Table 1:** Descriptive Statistics of Sample Characteristics.

Variable	Category	Frequency	Percentage	Variable	Category	Frequency	Percentage
Gender	Male	90	65.2%	Region	Beijing	52	37.7%
	Female	48	34.8%		Tianjin	40	29.0%
Age	25 and below	14	10.1%		Hebei	32	23.2%
	26 ~ 30	27	19.6%		Liaoning	14	10.1%
	31 ~ 35	29	21.0%	Asset Scale	Less than 500,000	57	41.3%
	36 ~ 40	37	26.8%		500,000–999,999	31	22.5%
	41 and above	31	22.5%		1,000,000–4,999,999	33	23.9%
Education	High school	12	8.7%		5,000,000–9,999,999	9	6.5%
	Associate degree	41	29.7%		10,000,000 and above	8	5.8%
	Bachelor’s degree	75	54.3%	Firm Age	1 year	41	29.7%
	Master’s degree	8	5.8%		2 years	27	19.6%
	Doctor	2	1.4%		3 years	27	19.6%
Industry	Information transmission, software and technology services	47	34.1%		4 years	30	21.7%
	Wholesale and retail	16	11.6%		5 years	7	5.1%
	Others	75	54.3%		6 years	6	4.3%

Note: Sample size = 138.

To assess whether the data approximated a normal distribution, the skewness and kurtosis of each measurement variable were examined. Results indicated that the absolute values of skewness were all less than 2, and the absolute values of kurtosis were all below 5, suggesting that the data met the assumptions of normality.

Given that all data in this study were self-reported by respondents, it was necessary to address the potential issue of Common Method Bias (CMB). Several procedural remedies were implemented during the questionnaire design stage. First, objective items were included to balance subjective evaluations. Second, the introduction to the survey explicitly stated that the study was for academic purposes only, assured respondents of the confidentiality of their responses, and clarified that there were no right or wrong answers. These measures aimed to reduce social desirability bias and privacy-related distortions. Third, the overall questionnaire length was carefully controlled to minimize respondent fatigue.

To further test for CMB, Harman’s one-factor test was conducted. The Kaiser-Meyer-Olkin (KMO) measure was 0.700, with a chi-square value of 1219.624 (*df* = 666, *p* < 0.01). An unrotated exploratory factor analysis yielded 11 distinct factors, with the first factor accounting for only 21.392% of the total variance—well below the critical threshold of 40%. This result suggests that common method bias is not a serious concern in this study.

### 3.2. Measurement of variables

To ensure both reliability and construct validity, this study adopted well-established scales from the extant literature to measure all core variables. All items were anchored on a five-point Likert scale (1 = strongly disagree; 5 = strongly agree).

(1) Independent Variable: Analytical Cognitive Style.

Analytical cognitive style was assessed using a six-item scale adapted from Kirton’s [13] original inventory, with refinements informed by Carnabuci and Diószegi [42]. Representative items include: “I enjoy analyzing problems in depth” and “I tend to conduct detailed analysis before making decisions.”

(2) Dependent Variables: Business Model Innovation.

Drawing upon the measurement framework developed by Zott and Amit [25] and further refined based on the work of Yi et al. [43], we measured two distinct forms of business model innovation.

Efficiency-oriented BMI was measured via eight items, such as “Our business model reduces inventory costs for participating entities.”

Novelty-oriented BMI was also measured using eight items, including “Our business model incorporates new types of partners.”

(3) Moderating Variables: Entrepreneurial Self-Efficacy and Environmental Uncertainty.

Entrepreneurial self-efficacy was captured using four items derived from Forbes [33], with adaptations based on Nag et al. [44] and Covin et al. [45]. Illustrative items include: “To what extent do you recognize your ability to generate novel ideas?” and “To what extent do you believe in your strategic planning capabilities?”

Environmental uncertainty was assessed using a four-item scale developed by Jansen et al. [46] and refined based on Jahanshahi et al. [47]. Sample items include: “The local market environment is highly volatile,” and “Our customers regularly request new products and services.”

(4) Control Variables.

To enhance the robustness of the empirical analysis, we included a set of control variables encompassing both individual- and firm-level characteristics: entrepreneur’s gender, age, and educational background; firm age; geographic region; industry sector; and initial capital at the time of founding. These were entered as dummy variables in the regression models to control for potential confounding effects.

### 3.3. Reliability and validity assessment

Reliability was assessed using **Cronbach’s* α* coefficients. Results indicated acceptable levels of internal consistency across all constructs: analytical cognitive style (*α* = 0.696), efficiency – oriented BMI (*α* = 0.661), novelty-oriented BMI (*α* = 0.735), entrepreneurial self-efficacy (*α* = 0.757), and environmental uncertainty (*α* = 0.670). In confirmatory research within the social sciences, a Cronbach’s α of 0.7 or higher is generally considered ideal. More specifically, when the data are derived from established scales or questionnaires with well-designed items, a Cronbach’s α of 0.65 or above indicates basic internal consistency and renders the data acceptable for analysis. DeVellis [48] also suggests that α ≥ 0.65 is the minimum acceptable threshold. In this study, the Cronbach’s α coefficients for all variables exceed 0.65, indicating good internal consistency for all measurement scales.

To further evaluate scale quality, we examined corrected item-total correlations (CITC). For all constructs, CITC values exceeded the recommended cutoff of 0.50, and removing any item did not significantly improve the scale’s internal consistency. Thus, all measurement items were retained in the final analysis.

Construct validity was assessed through confirmatory factor analysis (CFA) using AMOS 24.0. The model fit indices for each construct demonstrated acceptable to strong fit levels:

Analytical Cognitive Style: *χ²/df* = 1.676, *RMSEA* = 0.080, *CFI* = 0.943, *TLI* = 0.936, *SRMR* = 0.074.

Efficiency-Oriented BMI: *χ²/df* = 1.549, *RMSEA* = 0.052, *CFI* = 0.957, *TLI* = 0.924, *SRMR* = 0.043.

Novelty-Oriented BMI: *χ²/df* = 1.312, *RMSEA* = 0.050, *CFI* = 0.938, *TLI* = 0.913, *SRMR* = 0.062.

Entrepreneurial Self-Efficacy: *χ²/df* = 1.846, *RMSEA* = 0.046, *CFI* = 1.000, *TLI* = 1.007, *SRMR* = 0.033.

Environmental Uncertainty: *χ²/df* = 2.113, *RMSEA* = 0.052, *CFI* = 0.933, *TLI* = 0.987, *SRMR* = 0.037.

All item loadings were statistically significant at the 1% level and exceeded the recommended threshold of 0.40, confirming that the constructs demonstrated satisfactory convergent validity.

## 4. Empirical testing and analysis of results

### 4.1. Correlation analysis

As shown in the correlation analysis (Table 2), the correlation coefficients among the key variables are relatively low, indicating that multicollinearity is unlikely to be a serious concern in this study. Variance Inflation Factor (VIF) tests will be conducted subsequently to further assess potential multicollinearity among the variables.

**Table 2:** Means, Standard Deviations, and Correlation Coefficients of Study Variables.

Category	1	2	3	4	5	6	7	8	9	10	11	12
1. Analytical Cognitive Style	1											
2. Efficiency-Oriented Business Model Innovation	0.346**	1										
3. Novelty-Oriented Business Model Innovation	0.341**	0.109*	1									
4. Self-Efficacy	0.657**	0.515**	0.568**	1								
5. Environmental Uncertainty	0.501**	0.354**	0.228**	0.369**	1							
6. Entrepreneur Gender	0.019	−0.023	−0.229**	−0.115	0.177*	1						
7. Entrepreneur Age	−0.112	−0.037	−0.003	−0.084	−0.049	−0.050	1					
8. Entrepreneur Educational Background	−0.126	−0.075	−0.033	−0.096	−0.083	−0.045	0.205*	1				
9. Firm Age	0.062	−0.064	−0.061	−0.042	−0.051	0.231**	0.213*	−0.081	1			
10. Industry	−0.001	0.021	0.034	−0.036	0.122	0.043	0.311**	0.064	−0.078	1		
11. Initial Asset Scale at Business Establishment	0.018	−0.017	0.069	0.135	−0.022	−0.146	0.049	0.130	0.019	−0.160	1	
12. Region	0.029	−0.144	−0.011	−0.004	−0.136	−0.154	0.049	0.368**	−0.123	0.009	0.472**	1
Mean	4.2452	3.9574	3.9139	4.1232	4.0670	0.6522	0.5072	0.6159	0.6884	0.3406	0.3623	0.3768
Standard Deviation	0.4749	0.4479	0.5346	0.5431	0.5783	0.4780	0.5018	0.4881	0.4648	0.4756	0.4824	0.4864

Note: Variables 1–5 are measured as mean scores. Variable 6: Male = 1; Variable 7: Entrepreneur age ≤ 35 = 1; Variable 8: Entrepreneur educational background (Bachelor’s degree or above) = 1; Variable 9: Firm age (1–3 years) = 1; Variable 10: Information transmission, software and information services industry = 1; Variable 11: Initial asset scale ≥ 1 million RMB = 1; Variable 12: Beijing = 1. p < 0.05; ** p < 0.1. Sample size = 138.

### 4.2. Hypothesis testing

Table 3 presents the regression analysis results. Model 1 examines the effects of control variables on the dependent variable, efficiency-oriented business model innovation. Model 2 extends Model 1 by including the independent variable, analytical cognitive style. The maximum Variance Inflation Factor (VIF) values for all models are well below 10, indicating that multicollinearity is not a serious concern. The results of Model 2 indicate that analytical cognitive style is positively associated with efficiency-oriented BMI (*β* = 0.364, *p* < 0.01), thereby supporting Hypothesis 1a. Model 5 includes only the control variables predicting novelty-oriented business model innovation. Model 6 adds analytical cognitive style as the independent variable. The results of Model 6 show that analytical cognitive style significantly and positively affects novelty-oriented BMI (*β* = 0.355, *p* < 0.01), lending support to Hypothesis 1b.

**Table 3:** Regression Analysis Results.

	Efficiency-Oriented BMI				Novelty-Oriented BMI			
	Model 1	Model 2	Model 3	Model 4	Model 5	Model 6	Model 7	Model 8
Gender	−0.026	−0.026	0.069	−0.032	−0.231^***^	−0.231^***^	−0.214^***^	−0.302
Age	−0.025	0.021	0.007	0.010	−0.029	0.016	0.026	0.030
Educational Background	−0.022	0.025	0.045	0.044	−0.025	0.021	−0.002	−.0022
Firm Age	−0.076	−0.110	−0.092	−0.113	−0.009	−0.043	0.024	0.026
Industry	0.040	0.020	0.041	−0.005	0.068	0.049	0.040	0.036
Initial Asset Scale at Establishment	0.079	0.077	0.012	0.063	0.088	0.087	−0.029	0.061
Region	−0.185^*^	−0.218^*^	−0.186^**^	−0.190^*^	−0.080	−0.112	−0.006	−0.066
Analytical Cognitive Style		0.364^**^	−0.247**	0.433		0.355^***^	0.152^*^	0.189
Self-Efficacy			−0.280				0.248	
Analytical Cognitive Style ×Self-Efficacy			0.358^***^				−0.455^**^	
Environmental Uncertainty				0.300^*^				0.020^*^
Analytical Cognitive Style ×Environmental Uncertainty				0.089				−0.365^***^
*R* ^*2*^	0.034	0.161	0.330	0.218	0.064	0.184	0.393	0.258
*Adjusted R* ^*2*^	−0.018	0.109	0.277	0.157	0.013	0.134	0.345	0.200
*F*	0.657	3.104^***^	3.244^***^	3.547^**^	1.263	3.648^***^	3.816^***^	4.424^***^
*VIF(max)*	1.207	1.233	2.705	2.556	1.207	1.233	2.705	2.556

Note: Sample size = 138; * p < 0.1, ** p < 0.05, *** p < 0.01.

**Fig 1:**
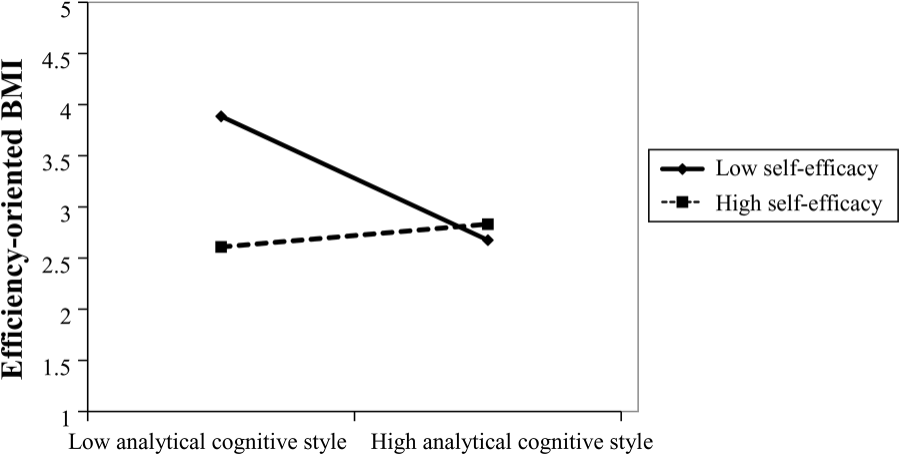
The Moderating Effect of Self-Efficacy on the Relationship Between Analytical Cognitive Style and Efficiency-Oriented Business Model Innovation.

Model 3 builds upon Model 2 by introducing the moderating variable—entrepreneurial self-efficacy—and the interaction term between analytical cognitive style and self-efficacy. The regression results reveal a significant interaction effect. Specifically, the interaction term is positive and significant (*β* = 0.358, *p* < 0.01), indicating that self-efficacy is positively associated with analytical cognitive style on efficiency-oriented BMI. In other words, when entrepreneurs exhibit high levels of self-efficacy, the positive impact of analytical reasoning on efficiency-oriented innovation is amplified (as illustrated in Fig 1). Therefore, Hypothesis 2a is supported.

However, Fig 1 also reveals a more nuanced pattern beyond the regression results. Specifically, when entrepreneurial self-efficacy reaches a very high level, the marginal effect of analytical cognitive style becomes less pronounced. This suggests that high self-efficacy may act as a compensatory mechanism, buffering against the disadvantages of a lower analytical cognitive style and enabling entrepreneurs to achieve efficiency-oriented innovations even without strongly analytical tendencies.

**Fig 2:**
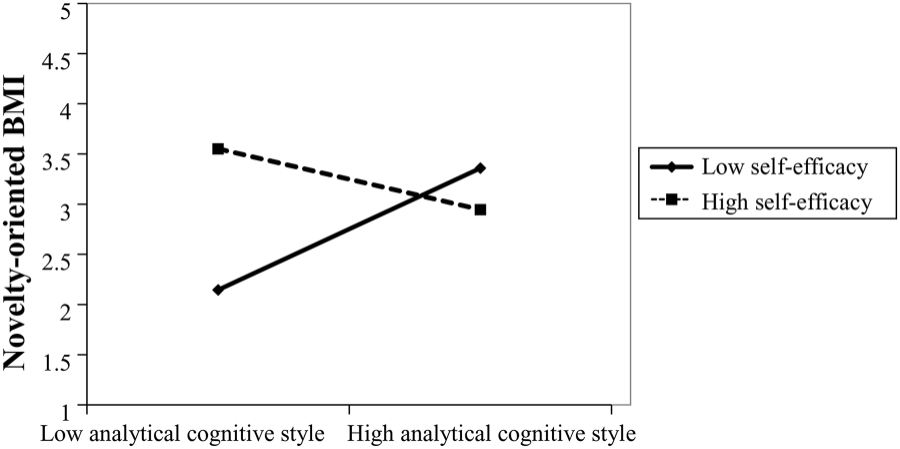
The Moderating Effect of Self-Efficacy on the Relationship Between Analytical Cognitive Style and Novelty-Oriented Business Model Innovation.

Model 7 expands upon Model 6 by incorporating self-efficacy and its interaction with analytical cognitive style. The results again show a significant moderating effect. The interaction coefficient is negative and significant (*β* = –0.455, *p* < 0.05), suggesting that self-efficacy weakens the positive relationship between analytical cognitive style and novelty-oriented BMI. That is, when self-efficacy is high, the positive influence of analytical cognition on novelty-oriented innovation diminishes (see Fig 2), thus supporting Hypothesis 2b.

**Fig 3:**
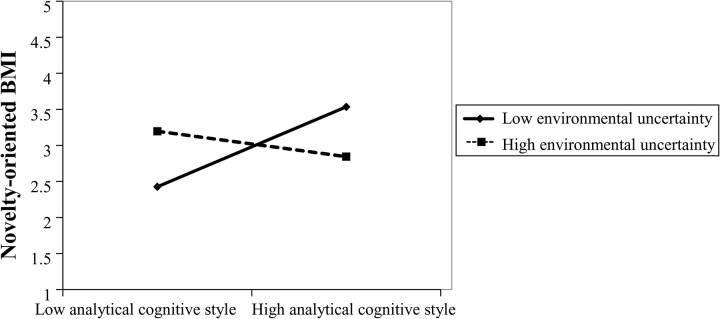
The Moderating Effect of Environmental Uncertainty on the Relationship Between Analytical Cognitive Style and Novelty-Oriented Business Model Innovation.

Model 4 includes environmental uncertainty and its interaction with analytical cognitive style based on Model 2. The results indicate that the interaction term is not statistically significant, suggesting no moderating effect on efficiency-oriented BMI. Hence, Hypothesis 3a is not supported. Model 8 builds upon Model 6 by including environmental uncertainty and its interaction with analytical cognitive style. The interaction effect is statistically significant and negative (*β* = –0.365, *p* < 0.01), implying that environmental uncertainty negatively moderates the relationship between analytical cognitive style and novelty-oriented BMI. Specifically, under conditions of high environmental uncertainty, the positive influence of analytical reasoning on novelty-oriented innovation is weakened (as depicted in Fig 3). Therefore, Hypothesis 3b is supported.

### 4.3. Robustness tests

#### 4.3.1. Subsample analysis based on alternative firm age criteria.

Conducting regression analyses on subsamples extracted from the original dataset is a widely used approach to assess the robustness of empirical results. While this study follows Zahra et al. [40] in defining new ventures as firms no older than six years, some researchers have adopted a stricter criterion, defining new ventures as firms that are four years old or younger [49]. To test the sensitivity of our findings to alternative definitions, we excluded firms that were five or six years old and re-ran the regression analyses using the remaining 125 valid observations.

The robustness results based on this restricted sub-sample are presented in Table 4. The findings show that Hypotheses 1a, 1b, 2a, 2b, and 3b remain statistically significant with consistent directions of effect, whereas Hypothesis 3a remains unsupported. These results confirm that the primary findings are robust to alternative definitions of new ventures.

**Table 4:** Robustness Tests of Results Using Selected Sample.

	Efficiency-Oriented BMI				Novelty-Oriented BMI			
	Model 1	Model 2	Model 3	Model 4	Model 5	Model 6	Model 7	Model 8
Gender	−0.062	−0.084	−0.018	−0.119	−0.178	−0.206^**^	−0.217^**^	−0.275^***^
Age	−0.038	0.053	0.031	0.018	−0.053	0.062	0.058	0.056
Educational Background	−0.072	−0.059	−0.023	−0.036	−0.034	−0.018	0.013	0.002
Firm Age	−0.006	−0.078	−0.055	−0.020	0.085	−0.007	0.021	0.019
Industry	0.101	0.074	0.084	0.055	0.057	0.023	0.000	−0.017
Initial Asset Scale at Establishment	−0.051	−0.052	−0.086	−0.034	−0.046	−0.048	−0.115	−0.079
Region	−0.006	−0.064	−0.097	−0.057	0.178	0.105	0.110	0.162
Analytical Cognitive Style		0.356^***^	0.056	0.190		0.450^***^	−0.169	0.100
Self-Efficacy			0.650^***^				0.583^***^	
Analytical Cognitive Style ×Self-Efficacy			0.229^*^				−0.292^**^	
Environmental Uncertainty				0.307^**^				0.098
Analytical Cognitive Style ×Environmental Uncertainty				0.025				−0.408^***^
*R* ^*2*^	0.020	0.134	0.336	0.192	0.068	0.250	0.490	0.332
*Adjusted R* ^*2*^	−0.072	0.040	0.243	0.080	−0.019	0.169	0.419	0.239
*F*	0.215	1.425^*^	3.639^***^	1.709^*^	0.784	3.088^***^	6.916^***^	3.581^***^
*VIF(max)*	1.260	1.286	2.053	2.168	1.260	1.286	2.053	2.168

Note: Sample size = 125; * p < 0.1, ** p < 0.05, *** p < 0.01.

#### 4.3.2. Alternative measurement approach for key variables.

Another common robustness approach involves re-specifying key constructs—typically the independent or dependent variables—using alternative measurement schemes [7]. To this end, we re-estimated our models using alternative measures of business model innovation (BMI). While the present study utilized an extended version of the Zott and Amit [25] scale, many prior studies have employed abbreviated forms of this instrument.

Accordingly, we followed the approach of Najafi-Tavani et al. [50] and shortened the original eight-item scales for efficiency- and novelty-oriented BMI by removing two items from each. We then recalculated composite indices using the remaining six items and re-ran the regression analyses. As shown in Table 5, the revised models yield results that are highly consistent with those of the primary analyses. All supported hypotheses remain significant, and the direction of effects remains unchanged. These findings further affirm the robustness of the study’s conclusions.

**Table 5:** Robustness Tests of Results by Replacing Key Variable Measurement Methods.

	Efficiency-Oriented BMI				Novelty-Oriented BMI			
	Model 1	Model 2	Model 3	Model 4	Model 5	Model 6	Model 7	Model 8
Gender	−0.031	−0.056	0.010	−0.104	−0.161	−0.188^*^	−0.188^**^	−0.262^***^
Age	0.021	0.080	0.046	0.049	−0.051	0.014	0.028	0.019
Educational Background	−0.058	−0.029	0.001	−0.001	−0.054	−0.023	−0.007	−0.004
Firm Age	−0.108	−0.148	−0.104	−0.103	0.018	−0.027	−0.008	−0.009
Industry	0.020	0.017	0.043	−0.011	0.015	0.011	−0.017	−0.046
Initial Asset Scale at Establishment	−0.138	−0.156	−0.180^*^	−0.148	−0.057	−0.078	−0.120	−0.098
Region	0.019	−0.021	−0.046	−0.006	0.186^*^	0.142	0.131	0.185^*^
Analytical Cognitive Style		0.378^***^	0.066	0.224		0.418^***^	−0.119	0.077
Self-Efficacy			0.591^***^				0.482^***^	
Analytical Cognitive Style ×Self-Efficacy			0.175^*^				−0.289^**^	
Environmental Uncertainty				0.298^**^				0.166
Analytical Cognitive Style ×Environmental Uncertainty				0.023				−0.358^***^
*R* ^*2*^	0.032	0.167	0.329	0.221	0.063	0.229	0.405	0.308
*Adjusted R* ^*2*^	−0.049	0.086	0.246	0.125	−0.015	0.154	0.332	0.222
*F*	0.392	2.075^**^	3.963^***^	2.303^**^	0.811	3.077^***^	5.521^***^	3.599^***^
*VIF(max)*	1.254	1.257	2.544	2.413	1.254	1.257	2.544	2.413

Note: Sample size = 138; * p < 0.1, ** p < 0.05, *** p < 0.01.

## 5. Discussion and conclusion

### 5.1. Conclusions of the study

In the current digital economy, data has become a critical asset in entrepreneurial decision-making. However, leveraging the full value of data requires not only high-quality data accumulation but also entrepreneurs’ enhanced cognitive capacity to interpret and utilize it effectively. Entrepreneurs with an analytical cognitive style place strong emphasis on precision and rigorous data analysis, which facilitates business model innovation (BMI) in data-intensive environments. This view is increasingly recognized in both theoretical and practical domains, and the empirical results of this study lend further support.

However, the positive relationship between analytical cognitive style and business model innovation is conditional, as it is influenced by characteristics of both the entrepreneur and the entrepreneurial environment. Building on prior research, this study examines the moderating effects of entrepreneurs’ self-efficacy and environmental uncertainty on the relationship between analytical cognitive style and business model innovation. The following conclusions are drawn:

On the one hand, entrepreneurs’ self-efficacy positively moderates the relationship between analytical cognitive style and efficiency-oriented business model innovation. When facing efficiency-oriented innovations, which tend to follow more predictable patterns, entrepreneurs with higher self-efficacy are more likely to trust analytical conclusions derived from data and, based on their capabilities, make decisions that promote efficiency-oriented innovation. Conversely, self-efficacy negatively moderates the effect of analytical style on novelty-oriented BMI. In uncertain and novel innovation scenarios, overconfidence may lead high self-efficacy entrepreneurs to question ambiguous or non-definitive data insights, thereby reducing the likelihood of pursuing disruptive innovation.

On the other hand, environmental uncertainty negatively moderates the effect of analytical cognitive style on novelty-oriented BMI. Novel business models often involve unproven value propositions and high ambiguity, which cannot be resolved solely through logical reasoning or quantitative analysis. In such contexts, heightened environmental uncertainty amplifies perceived risks, reducing entrepreneurs’ willingness to engage in exploratory business model experimentation. Although a positive moderating effect of environmental uncertainty was theorized for efficiency-oriented BMI, empirical results did not support this relationship.

### 5.2. Theoretical contributions

This study contributes to the entrepreneurship literature in two key ways:

First, it sheds light on the differentiated impact of analytical cognitive style on two distinct types of BMI. Previous cognitive studies have rarely distinguished between efficiency- and novelty-oriented innovations, potentially overlooking their divergent mechanisms. This study uncovers a non-symmetric “facilitation–inhibition” pattern—where analytical cognition promotes efficiency-oriented innovation while potentially constraining novelty-driven efforts—thereby enriching the theoretical understanding of the cognitive foundations of BMI.

Second, by integrating social cognitive theory and contingency theory, the study proposes a conceptual framework linking cognitive style, contextual factors, and innovation outcomes. It highlights how entrepreneurial self-efficacy and environmental uncertainty serve as boundary conditions that shape the efficacy of cognitive strategies in innovation contexts. This enhances our understanding of individual–environment alignment in entrepreneurial cognition.

### 5.3. Practical implications

On the one hand, entrepreneurs should develop a “cognitive alignment” mindset that is compatible with the digital and intelligent economy. Research indicates that analytical cognitive style has a significant positive effect on different types of business model innovation. Therefore, under a digitalized context, entrepreneurs and their teams should avoid over-reliance on intuitive decision-making. They should actively learn statistical knowledge and data analysis methods, emphasizing the application of analytical cognitive styles. Building on this cognitive style, entrepreneurs can cultivate data awareness, enhance data literacy, and promote business model innovation.

Entrepreneurs can also leverage mature e-commerce platforms or establish their own data platforms to collect, integrate, process, and analyze data, thereby maximizing its value to support entrepreneurial activities. For example, by connecting to or building platforms, entrepreneurs can analyze user purchasing behavior to enable precise product recommendations, monitor inventory and sales in real time, optimize supply chain management, and reduce inventory costs.

On the other hand, entrepreneurs should cultivate “situational sensitivity” as a dynamic capability. Environmental uncertainty significantly influences the effectiveness of cognitive styles. New ventures should establish robust environmental monitoring systems to regularly assess market trends. In highly uncertain environments, they should prioritize efficiency-oriented business model innovation, focusing on optimizing existing processes to enhance competitiveness. In relatively stable environments, greater investment in novelty-oriented innovation is appropriate.

Furthermore, for entrepreneurs with high self-efficacy, it is important to recognize the limitations of their knowledge and experience when pursuing novelty-oriented business model innovation. Given the complexity and difficulty of such innovations, neglecting information collection and acquisition may lead to failure. Therefore, highly self-efficacious entrepreneurs should emphasize knowledge accumulation and information gathering when engaging in high-innovation decision-making, demonstrating meticulous thinking, attention to detail, and logical clarity to achieve disruptive innovation.

Overall, to succeed in the digital era, entrepreneurs must learn to make data-driven decisions while remaining sensitive to environmental changes. Decision-making should not rely solely on intuition; entrepreneurs need to develop data analysis skills and leverage platform tools to understand user behavior.

At the same time, they must respond flexibly to market dynamics. In complex and uncertain environments, the initial focus should be on improving efficiency and streamlining processes, whereas in more stable conditions, entrepreneurs can more boldly experiment with novel business models. Even highly confident entrepreneurs should continuously learn and gather information to avoid mistakes stemming from insufficient experience. Only by integrating “data-driven decision-making” with “adaptive responsiveness” can entrepreneurs navigate innovation more steadily and effectively.

### 5.4. Limitations and future research

This study has several limitations that warrant further investigation in future research. First, the use of self-reported questionnaires may introduce measurement errors due to respondents’ misunderstandings or biases, and the cross-sectional design limits the ability to draw causal inferences. Future studies should consider expanding survey channels, providing more thorough respondent training, and incorporating additional data sources, such as publicly available panel data. In particular, conducting annual surveys on the same sample firms would allow for the collection of time-series data, enabling analysis of the causal relationship between analytical cognitive style and business model innovation.

Second, the analysis primarily focuses on firms in four Chinese provinces, which may limit the generalizability of the findings due to the restricted geographic scope and sample size. Future research should broaden the survey regions and increase the sample size. Collecting data from firms in different countries or regions would also allow for cross-cultural comparisons, thereby enriching insights and enhancing the external validity of the findings.

Third, this study empirically examined only two representative moderating factors—entrepreneurs’ self-efficacy and environmental uncertainty—on the relationship between analytical cognitive style and business model innovation. Future research should explore additional entrepreneurial traits, such as emotional resilience and grit, as well as environmental characteristics, including dynamism and complexity, to gain a more comprehensive understanding of the boundary conditions influencing this relationship.

## Supporting information

S1 File Supporting Information. (DOC)
