# Supplementary material for: The impact of analytical cognitive style on business model innovation in new ventures: The moderating role of self-efficacy and environmental uncertainty
Source: PLoS One. 2025 Oct 24;20(10):e0335256. doi: 10.1371/journal.pone.0335256 (PMC12551833; doi:10.1371/journal.pone.0335256)
Supplement: S1 File — (DOC) [file pone.0335256.s001.doc]

Table 1 Descriptive Statistics of Sample Characteristics

| Variable | Category | Frequency | Percentage | Variable | Category | Frequency | Percentage |
| --- | --- | --- | --- | --- | --- | --- | --- |
| Gender | Male | 90 | 65.2% | Region | Beijing | 52 | 37.7% |
|  | Female | 48 | 34.8% |  | Tianjin | 40 | 29.0% |
| Age | 25 and below | 14 | 10.1% |  | Hebei | 32 | 23.2% |
|  | 26~30 | 27 | 19.6% |  | Liaoning | 14 | 10.1% |
|  | 31~35 | 29 | 21.0% | Asset Scale | Less than 500,000 | 57 | 41.3% |
|  | 36~40 | 37 | 26.8% |  | 500,000–999,999 | 31 | 22.5% |
|  | 41 and above | 31 | 22.5% |  | 1,000,000–4,999,999 | 33 | 23.9% |
| Education | High school | 12 | 8.7% |  | 5,000,000–9,999,999 | 9 | 6.5% |
|  | Associate degree | 41 | 29.7% |  | 10,000,000 and above | 8 | 5.8% |
|  | Bachelor’s degree | 75 | 54.3% | Firm Age | 1 year | 41 | 29.7% |
|  | Master’s degree | 8 | 5.8% |  | 2 years | 27 | 19.6% |
|  | Doctor | 2 | 1.4% |  | 3 years | 27 | 19.6% |
| Industry | Information transmission, software and technology services | 47 | 34.1% |  | 4 years | 30 | 21.7% |
|  | Wholesale and retail | 16 | 11.6% |  | 5 years | 7 | 5.1% |
|  | Others | 75 | 54.3% |  | 6 years | 6 | 4.3% |

Note: Sample size = 138.

Table 2 Means, Standard Deviations, and Correlation Coefficients of Study Variables

| Category | 1 | 2 | 3 | 4 | 5 | 6 | 7 | 8 | 9 | 10 | 11 | 12 |
| --- | --- | --- | --- | --- | --- | --- | --- | --- | --- | --- | --- | --- |
| 1.Analytical Cognitive Style | 1 |  |  |  |  |  |  |  |  |  |  |  |
| 2.Efficiency-Oriented Business Model Innovation | 0.346** | 1 |  |  |  |  |  |  |  |  |  |  |
| 3.Novelty-Oriented Business Model Innovation | 0.341** | 0.109* | 1 |  |  |  |  |  |  |  |  |  |
| 4.Self-Efficacy | 0.657** | 0.515** | 0.568** | 1 |  |  |  |  |  |  |  |  |
| 5.Environmental Uncertainty | 0.501** | 0.354** | 0.228** | 0.369** | 1 |  |  |  |  |  |  |  |
| 6.Entrepreneur Gender | 0.019 | -0.023 | -0.229** | -0.115 | 0.177* | 1 |  |  |  |  |  |  |
| 7.Entrepreneur Age | -0.112 | -0.037 | -0.003 | -0.084 | -0.049 | -0.050 | 1 |  |  |  |  |  |
| 8.Entrepreneur Educational Background | -0.126 | -0.075 | -0.033 | -0.096 | -0.083 | -0.045 | 0.205* | 1 |  |  |  |  |
| 9.Firm Age | 0.062 | -0.064 | -0.061 | -0.042 | -0.051 | 0.231** | 0.213* | -0.081 | 1 |  |  |  |
| 10.Industry | -0.001 | 0.021 | 0.034 | -0.036 | 0.122 | 0.043 | 0.311** | 0.064 | -0.078 | 1 |  |  |
| 11.Initial Asset Scale at Business Establishment | 0.018 | -0.017 | 0.069 | 0.135 | -0.022 | -0.146 | 0.049 | 0.130 | 0.019 | -0.160 | 1 |  |
| 12.Region | 0.029 | -0.144 | -0.011 | -0.004 | -0.136 | -0.154 | 0.049 | 0.368** | -0.123 | 0.009 | 0.472** | 1 |
| Mean | 4.2452 | 3.9574 | 3.9139 | 4.1232 | 4.0670 | 0.6522 | 0.5072 | 0.6159 | 0.6884 | 0.3406 | 0.3623 | 0.3768 |
| Standard Deviation | 0.4749 | 0.4479 | 0.5346 | 0.5431 | 0.5783 | 0.4780 | 0.5018 | 0.4881 | 0.4648 | 0.4756 | 0.4824 | 0.4864 |

Note: Variables 1–5 are measured as mean scores. Variable 6: Male = 1; Variable 7: Entrepreneur age ≤ 35 = 1; Variable 8: Entrepreneur educational background (Bachelor’s degree or above) = 1; Variable 9: Firm age (1–3 years) = 1; Variable 10: Information transmission, software and information services industry = 1; Variable 11: Initial asset scale ≥ 1 million RMB = 1; Variable 12: Beijing = 1. p < 0.05; ** p < 0.1. Sample size = 138.

Table 3 Regression Analysis Results

|  | Efficiency-Oriented BMI | | | | Novelty-Oriented BMI | | | |
| --- | --- | --- | --- | --- | --- | --- | --- | --- |
|  | Model 1 | Model 2 | Model 3 | Model 4 | Model 5 | Model 6 | Model 7 | Model 8 |
| Gender | -0.026 | -0.026 | 0.069 | -0.032 | -0.231^***^ | -0.231^***^ | -0.214^***^ | -0.302 |
| Age | -0.025 | 0.021 | 0.007 | 0.010 | -0.029 | 0.016 | 0.026 | 0.030 |
| Educational Background | -0.022 | 0.025 | 0.045 | 0.044 | -0.025 | 0.021 | -0.002 | -.0022 |
| Firm Age | -0.076 | -0.110 | -0.092 | -0.113 | -0.009 | -0.043 | 0.024 | 0.026 |
| Industry | 0.040 | 0.020 | 0.041 | -0.005 | 0.068 | 0.049 | 0.040 | 0.036 |
| Initial Asset Scale at Establishment | 0.079 | 0.077 | 0.012 | 0.063 | 0.088 | 0.087 | -0.029 | 0.061 |
| Region | -0.185^*^ | -0.218^*^ | -0.186^**^ | -0.190^*^ | -0.080 | -0.112 | -0.006 | -0.066 |
| Analytical Cognitive Style |  | 0.364^**^ | -0.247** | 0.433 |  | 0.355^***^ | 0.152^*^ | 0.189 |
| Self-Efficacy |  |  | -0.280 |  |  |  | 0.248 |  |
| Analytical Cognitive Style ×Self-Efficacy |  |  | 0.358^***^ |  |  |  | -0.455^**^ |  |
| Environmental Uncertainty |  |  |  | 0.300^*^ |  |  |  | 0.020^*^ |
| Analytical Cognitive Style ×Environmental Uncertainty |  |  |  | 0.089 |  |  |  | -0.365^***^ |
| *R^2^* | 0.034 | 0.161 | 0.330 | 0.218 | 0.064 | 0.184 | 0.393 | 0.258 |
| *Adjusted R^2^* | -0.018 | 0.109 | 0.277 | 0.157 | 0.013 | 0.134 | 0.345 | 0.200 |
| *F* | 0.657 | 3.104^***^ | 3.244^***^ | 3.547^**^ | 1.263 | 3.648^***^ | 3.816^***^ | 4.424^***^ |
| *VIF(max)* | 1.207 | 1.233 | 2.705 | 2.556 | 1.207 | 1.233 | 2.705 | 2.556 |

Note: Sample size = 138; * p < 0.1, ** p < 0.05, *** p < 0.01.

Table 4. Robustness Tests of Results Using Selected Sample

|  | Efficiency-Oriented BMI | | | | Novelty-Oriented BMI | | | |
| --- | --- | --- | --- | --- | --- | --- | --- | --- |
|  | Model 1 | Model 2 | Model 3 | Model 4 | Model 5 | Model 6 | Model 7 | Model 8 |
| Gender | -0.062 | -0.084 | -0.018 | -0.119 | -0.178 | -0.206^**^ | -0.217^**^ | -0.275^***^ |
| Age | -0.038 | 0.053 | 0.031 | 0.018 | -0.053 | 0.062 | 0.058 | 0.056 |
| Educational Background | -0.072 | -0.059 | -0.023 | -0.036 | -0.034 | -0.018 | 0.013 | 0.002 |
| Firm Age | -0.006 | -0.078 | -0.055 | -0.020 | 0.085 | -0.007 | 0.021 | 0.019 |
| Industry | 0.101 | 0.074 | 0.084 | 0.055 | 0.057 | 0.023 | 0.000 | -0.017 |
| Initial Asset Scale at Establishment | -0.051 | -0.052 | -0.086 | -0.034 | -0.046 | -0.048 | -0.115 | -0.079 |
| Region | -0.006 | -0.064 | -0.097 | -0.057 | 0.178 | 0.105 | 0.110 | 0.162 |
| Analytical Cognitive Style |  | 0.356^***^ | 0.056 | 0.190 |  | 0.450^***^ | -0.169 | 0.100 |
| Self-Efficacy |  |  | 0.650^***^ |  |  |  | 0.583^***^ |  |
| Analytical Cognitive Style ×Self-Efficacy |  |  | 0.229^*^ |  |  |  | -0.292^**^ |  |
| Environmental Uncertainty |  |  |  | 0.307^**^ |  |  |  | 0.098 |
| Analytical Cognitive Style ×Environmental Uncertainty |  |  |  | 0.025 |  |  |  | -0.408^***^ |
| *R^2^* | 0.020 | 0.134 | 0.336 | 0.192 | 0.068 | 0.250 | 0.490 | 0.332 |
| *Adjusted R^2^* | -0.072 | 0.040 | 0.243 | 0.080 | -0.019 | 0.169 | 0.419 | 0.239 |
| *F* | 0.215 | 1.425^*^ | 3.639^***^ | 1.709^*^ | 0.784 | 3.088^***^ | 6.916^***^ | 3.581^***^ |
| *VIF(max)* | 1.260 | 1.286 | 2.053 | 2.168 | 1.260 | 1.286 | 2.053 | 2.168 |

Note: Sample size = 125; * p < 0.1, ** p < 0.05, *** p < 0.01.

Table 5. Robustness Tests of Results by Replacing Key Variable Measurement Methods

|  | Efficiency-Oriented BMI | | | | Novelty-Oriented BMI | | | |
| --- | --- | --- | --- | --- | --- | --- | --- | --- |
|  | Model 1 | Model 2 | Model 3 | Model 4 | Model 5 | Model 6 | Model 7 | Model 8 |
| Gender | -0.031 | -0.056 | 0.010 | -0.104 | -0.161 | -0.188^*^ | -0.188^**^ | -0.262^***^ |
| Age | 0.021 | 0.080 | 0.046 | 0.049 | -0.051 | 0.014 | 0.028 | 0.019 |
| Educational Background | -0.058 | -0.029 | 0.001 | -0.001 | -0.054 | -0.023 | -0.007 | -0.004 |
| Firm Age | -0.108 | -0.148 | -0.104 | -0.103 | 0.018 | -0.027 | -0.008 | -0.009 |
| Industry | 0.020 | 0.017 | 0.043 | -0.011 | 0.015 | 0.011 | -0.017 | -0.046 |
| Initial Asset Scale at Establishment | -0.138 | -0.156 | -0.180^*^ | -0.148 | -0.057 | -0.078 | -0.120 | -0.098 |
| Region | 0.019 | -0.021 | -0.046 | -0.006 | 0.186^*^ | 0.142 | 0.131 | 0.185^*^ |
| Analytical Cognitive Style |  | 0.378^***^ | 0.066 | 0.224 |  | 0.418^***^ | -0.119 | 0.077 |
| Self-Efficacy |  |  | 0.591^***^ |  |  |  | 0.482^***^ |  |
| Analytical Cognitive Style ×Self-Efficacy |  |  | 0.175^*^ |  |  |  | -0.289^**^ |  |
| Environmental Uncertainty |  |  |  | 0.298^**^ |  |  |  | 0.166 |
| Analytical Cognitive Style ×Environmental Uncertainty |  |  |  | 0.023 |  |  |  | -0.358^***^ |
| *R^2^* | 0.032 | 0.167 | 0.329 | 0.221 | 0.063 | 0.229 | 0.405 | 0.308 |
| *Adjusted R^2^* | -0.049 | 0.086 | 0.246 | 0.125 | -0.015 | 0.154 | 0.332 | 0.222 |
| *F* | 0.392 | 2.075^**^ | 3.963^***^ | 2.303^**^ | 0.811 | 3.077^***^ | 5.521^***^ | 3.599^***^ |
| *VIF(max)* | 1.254 | 1.257 | 2.544 | 2.413 | 1.254 | 1.257 | 2.544 | 2.413 |

Note: Sample size = 138; * p < 0.1, ** p < 0.05, *** p < 0.01.

We confirm that our submission contains the “minimal data set” as defined by PLOS ONE. The data supporting the conclusions of the manuscript have been provided as **Supporting Information files**. These include:

The raw values underlying all reported means, standard deviations, and other descriptive statistics;

The data points used to construct all figures and graphs;

Key variables extracted from the survey responses.

To protect the confidentiality of participating enterprises, all data have been **anonymized and de-identified**, and no identifiable information (such as company names or contact details) is included.

As all data are already provided as Supporting Information, no additional repository upload is required. For any further requests regarding access to the data, interested parties may contact the corresponding author directly.
